# Editorial: Human Molecular and Physiological Responses to Hypoxia

**DOI:** 10.3389/fphys.2022.888005

**Published:** 2022-04-29

**Authors:** Sandro Malacrida, Giacomo Strapazzon, Simona Mrakic-Sposta, Matiram Pun, Annalisa Cogo

**Affiliations:** ^1^ Institute of Mountain Emergency Medicine, Eurac Research, Bolzano, Italy; ^2^ Institute of Clinical Physiology—National Research Council IFC—CNR, Milan, Italy; ^3^ Department of Physiology and Pharmacology, Cumming School of Medicine, University of Calgary, Calgary, AB, Canada; ^4^ University of Ferrara, Ferrara, Italy; ^5^ Center for Exercise and Sport Science University of Ferrara, Ferrara, Italy; ^6^ Institute Pio XII, Misurina, Italy

**Keywords:** hypoxia, altitude, blood, hypobaric, physiological, acclimatization, molecular, cognition

Towards the end of the 19th century, the French physician Denis Jourdan was the first to understand and state the critical role of the reduction of oxygen at altitude, which he defined as anoxemia. This term indicated the diminished quantity of oxygen contained in the blood of people living at high altitude, where the tension of the oxygen in the surrounding air is considerably decreased (West and Richalet, 2013). In the following 150 years, studies on hypoxia took off, ranging from purely clinical and functional aspects to cellular and biomolecular ones, from acute to chronic hypoxia and analyzing not only the altitude-hypoxia but also the hypoxia related to underlying diseases. Currently, the study of pathophysiological responses at altitude is a model to investigate the mechanisms of response to hypoxia in any condition, also in critical illnesses (Grocott et al., 2007).

In this special issue, a series of ten articles with different approaches applied to the study of molecular and physiological responses to hypoxia were collected.

A crucial question is the effect of prolonged hypoxia on circulating plasma lipid profile, more specifically its capacity to increase plasma triglyceride (TG) concentrations. In animal models, hypoxia has been shown to have detrimental effects on many aspects of triglyceride metabolism. Rene Morin et al. wrote a mini review highlighting that hypoxia tends to negatively affect TG levels by increasing the concentration of denser triglyceride-rich lipoproteins, mainly in prandial and postprandial states. These results can help to develop strategies to mitigate the effect of hypoxia on TG levels and the burden of a possible increase of cardiovascular risk. Ortiz-Prado et al. in fact, compared two populations with similar genetical, sociodemographic and economical characteristic, but living at different altitudes. They could, therefore, focus on the difference given by the altitude. They reconfirmed the well-known adaptive physiological changes related to life at altitude and they reported clinical differences in the plasma lipid profile, with higher levels of cholesterol and high density and low-density lipoproteins in Andinean population versus the Amazonian-basin one. The 10-years risk for cardiovascular disease was not different between the two groups. Pooja Kumarie et al. investigated the response to long-term high-altitude exposure on physiological indices, pro-inflammatory cytokines (IL-6, TNFα and CRP) and plasma proteome in 105 healthy male military subjects at sea level and after a short (7 days) at 3,520 m, and a long-term stay (3 months) at 4,176 m. Plasma proteomics studies revealed higher levels of apolipoproteins (APOB, APOCI, APOCIII, APOE, and APOL), carbonic anhydrases (CA1 and CA2) and proinflammatory cytokines during hypoxia exposure. These results suggest a vascular inflammation and demonstrate that long-term stay at high altitude exacerbate dyslipidemia and associated disorders.

Effects of hypoxia on different organs and populations has been investigated in different studies. Pedreros-Lobos et al. studied a group of miners at sea level and at moderate altitude. Despite the well-known decrease of VO_2_ max at altitude, they found that work capacity, heart rate, and ventilation did not change, suggesting that work efficiency was maintained. They observed a higher prevalence of overweight and obesity, as well as sedentarism in all miner populations: there was an increase in cardiometabolic risk unrelated to altitude, despite those markers of inflammation like hsCRP were increased at moderate altitudes. Fan et al. studied muscular and cerebral tissue oxygenation using near-infrared spectrometry (NIRS) in ten Han Chinese and ten Tibetan subjects during incremental cycling to exhaustion in normobaric normoxia and in hypobaric hypoxia simulating 5,000 m. The results showed a higher brain tissue oxygenation in Tibetan subjects compared to Han Chinese ones during maximal exercise in normoxia, but lower muscle tissue oxygenation during exercise in hypoxia. The authors conclude that Tibetan subjects seem to privilege oxygenation of the brain at the expense of that of the muscle. Functional effects of acute hypobaric hypoxia exposure on the brain were investigated by Falla et al. They reported a reduced processing speed in the first 24 h of exposure of lowlanders at 3,200 m that was quickly reversed after 36 h. These results demonstrate a cognitive impairment after acute exposure to altitude and the burden of an increased risk of accidents. Pernett et al. with an original and proved method studied the effect of 10 minutes acute normobaric hypoxia on spleen volume contraction. They showed a significant spleen volume contraction with Hb increase. This rapid spleen response is evident already after 3 min and can have a protective effect during the first minutes of sudden exposure to severe hypoxia. Sibonama et al. aimed to differentiate subjects suffering from acute mountain sickness (AMS) from those who do not, analyzing urine metabolites with nuclear magnetic resonance (NMR) based metabolomics. In this preliminary report they showed differences in the amount of creatine and acetylcarnitine (elevated), xanthine, hypoxanthine, and taurine (suppressed) in the urine between the subjects suffering from AMS vs. those who do not. They hypothesize that a metabolite profile at sea level could help in the screening for AMS susceptibility at altitude. Khalife et al. aimed to evaluate the possibility to induce erythropoietin (EPO), reticulocytes and hemoglobin stimulation in patients after surgery, avoiding the need for blood transfusions. They investigated the so-called NOP (Normobaric Oxygen Paradox) a physiologic mechanism of relative hypoxia after an exposure to hyperoxia, that induces an increase of endogenous erythropoietin (EPO) production. The comparison between a NOP-treated group and a control group did not show any difference on EPO increase, reticulocytes count and hemoglobin. Antonelli et al. investigated the expression of cytokines IL-6, IL-8, and VEGF (key mediators of the hematopoietic niche) *in vitro* in human macrophages and cell lines under anoxic conditions and after a treatment with oxygenated or deoxygenated red blood cells (RBCs). They found that the treatment with oxygenated RBCs up-regulates IL-8 mRNA, down-regulates IL-6 and VEGF expression in an HIF-1α independent mechanism in anoxic condition. This does not occur when deoxygenated RBCs are used. These are preliminary finding that can stimulate future research.

Despite the heterogeneity of the studies, this special issue highlights the importance of a better understanding of the responses to hypoxia and how our knowledge is still limited after nearly two centuries. An integrative approach between molecular and physiological measures should be fostered in future in in-field and simulated studies.
